# Effects of Sterilization Methods on Different 3D Printable Materials for Templates of Physician-Modified Aortic Stent Grafts Used in Vascular Surgery—A Preliminary Study

**DOI:** 10.3390/ijms23073539

**Published:** 2022-03-24

**Authors:** Paweł Rynio, Katarzyna Galant, Łukasz Wójcik, Bartłomiej Grygorcewicz, Arkadiusz Kazimierczak, Aleksander Falkowski, Piotr Gutowski, Barbara Dołęgowska, Miłosz Kawa

**Affiliations:** 1 Department of Vascular Surgery, Pomeranian Medical University in Szczecin, Powstańców Wielkopolskich 72, 70-111 Szczecin, Poland; biker2000@wp.pl (A.K.); piotr_gutowski@poczta.onet.pl (P.G.); 2 Department of Microbiology, Immunology and Laboratory Medicine, Pomeranian Medical University in Szczecin, Powstańców Wielkopolskich 72, 70-111 Szczecin, Poland; kasieg231@wp.pl (K.G.); b.grygorcewicz@gmail.com (B.G.); barbara.dolegowska@pum.edu.pl (B.D.); 3 Department of Radiology, Pomeranian Medical University in Szczecin, Powstańców Wielkopolskich 72, 70-111 Szczecin, Poland; woj.luka@gmail.com (Ł.W.); bakhis@hot.pl (A.F.); kawamilosz@gmail.com (M.K.)

**Keywords:** sterilization, 3D printing, aortic template, physician-modified stent graft, surgical guide

## Abstract

Three-dimensionally-printed aortic templates are increasingly being used to aid in the modification of stent grafts in the treatment of urgent, complex aortic disorders, often of an emergency nature. The direct contact between the aortic template and the stent graft implies the necessity of complete sterility. Currently, the efficacy of sterilizing aortic templates and the effect of sterilization on the geometry of tubular aortic models are unknown. A complex case of aortic arch dissection was selected to prepare a 3D-printed aortic arch template, which was then manufactured in six popular printing materials: polylactic acid (PLA), nylon, polypropylene (PP), polyethylene terephthalate glycol (PETG), and a rigid and flexible photopolymer resin using fused deposition modeling (FDM) and stereolithography (SLA). The 3D models were contaminated with *Geobacillus stearothermophilus* broth and *Bacillus atrophaeus.* The sterilization was performed using three different methods: heat (105 °C and 121 °C), hydrogen peroxide plasma, and ethylene oxide gas. Before and after sterilization, the aortic templates were scanned using computed tomography to detect any changes in their morphology by comparing the dimensions. All sterilization methods were effective in the elimination of microorganisms. Steam sterilization in an autoclave at 121 °C caused significant deformation of the aortic templates made of PLA, PETG, and PP. The other materials had stable geometries, and changes during mesh comparisons were found to be submillimeter. Similarly, plasma, gas, and heat at 105 °C did not change the shapes of aortic templates observed macroscopically and using mesh analysis. All mean geometry differences were smaller than 0.5 mm. All sterilization protocols tested in our study were equally effective in destroying microorganisms; however, differences occurred in the ability to induce 3D object deformation. Sterilization at high temperatures deformed aortic templates composed of PLA, PETG, and PP. This method was suitable for nylon, flexible, and rigid resin-based models. Importantly, plasma and gas sterilization were appropriate for all tested printing materials, including PLA, PETG, PP, nylon, flexible and rigid resins. Moreover, sterilization of all the printed models using our novel protocol for steam autoclaving at 105 °C was also 100% effective, which could represent a significant advantage for health centers, which can therefore use one of the most popular and cheap methods of medical equipment disinfection for the sterilization of 3D models as well.

## 1. Introduction

In recent years, the technique of implementing surgeon-modified stent grafts using specific 3D-printed aortic templates has gained prominence [1,2,3]. Patients with complex thoracic, abdominal, and thoracoabdominal aortic diseases could be treated with modified fenestrated aortic stent grafts [4]. These are indicated for patients with giant or symptomatic aneurysms who cannot wait for a custom-made fenestrated stent graft to be fabricated due to the significant risk of aneurysm rupture [4]. The use of three-dimensional aortic templates is thought to increase the quality and accuracy of the physician-modified fenestrated stent graft as a part of personalized medicine that is tailored with precision for a specific vascular problem in an exact patient. The surgeon applies the aortic 3D template under a sterile operating room setting to the standard aortic stent graft and marks the fenestration positions to ensure blood flow to aortic side branches [2]. This stage carries a risk of stent graft contamination. It is critical to follow aseptic principles and ensure that all surgical tools and aids are totally sterile. However, to the best of our knowledge, at the moment, there are no research reports on the efficacy of sterilization of these aortic 3D templates. Although three-dimensional printed aortic templates have been demonstrated to be quite accurate [5], the effect of sterilization on the 3D-printed aortic template’s geometry is also unclear, and it might be fatal if altered.

Sterilization of 3D-printed models has been accomplished by steam sterilization, gas sterilization, and plasma sterilization [6]. Steam sterilization is carried out in autoclaves under precisely controlled pressure, temperature, and time conditions. In practice, two temperatures are used: 121 degrees Celsius with a gravity autoclave and higher, 132 degrees Celsius with a vacuum autoclave, with minimum decontamination periods of 30 min and 4 min, respectively. Steam sterilization is less effective on complex objects, of which the surfaces are not directly in contact with the steam, as well as on porous objects. The usage of this technology is restricted with objects composed of heat-sensitive materials [6]. Therefore, low-temperature sterilization techniques may be appropriate for such objects. Sterilization with ethylene oxide (ETO) is performed between 37 and 63 degrees Celsius with 1–6 h saturation [6]. However, due to its carcinogenic properties, a further step of mechanical aeration is obligatorily required, in which harmful ETO residues are desorbed (it lasts 8–12 h at 50–60 °C). Gas sterilization efficiency may be decreased for items with lengthy and/or narrow channels, inorganic salts, and organic compounds [6]. Hydrogen peroxide in the plasma (HPP) state generates free radicals that disrupt microorganism metabolism [6]. The sterilizing cycle takes 28–52–73 min, depending on the type of device. Here, a decrease in decontamination efficiency is seen for the same factors as for gas sterilization.

The three-dimensional aortic template is fabricated using a 3D printing process [7]. The specific representation of the patient’s vascular anatomy is provided via segmentation of the computed tomography (CT) images obtained in the angiographic phase. The result of the segmentation is transformed into a 3D surface model, and then after the modeling stage it is transmitted to the 3D printer. Fused deposition modeling (FDM) and stereolithography (SLA) are the most widespread 3D printing technologies. FDM is based on melting the filament and layering it according to a computer-determined pattern [8]. In FDM technology, 3D vascular models have been created using polylactic acid (PLA), nylon, polyglycolic acid (PGA), poly-4-hydroxybutyrate, and acrylonitrile butadiene styrene (ABS) [7,9]. Stereolithography is an additive manufacturing process that uses a laser beam to cure a photosensitive resin layer-by-layer. Both technologies use mass-production materials, such as injection molding, which entails injecting plasticized material into a mold, solidifying and forming an object. Injection-molded objects have defined physical and chemical properties and, as a result, standardized sterilizing processes. The use of the same materials in additive manufacturing technology causes the deterioration of the mechanical properties of the created objects [10].

3D models of the vascular system are spatially complex objects containing narrow channels and wall surfaces facing their interior. Uneven surfaces characterize models made in rapid prototyping technologies due to their layered structure [10]. Grooves between the layers could reduce the effectiveness of bacterial decontamination methods. Another implication of the layered structure of 3D models is the deterioration of the physical properties of the models, i.e., the ability to retain their geometry when exposed to high temperature and chemical irritants [10]. In particular, the deformation of the 3D printed aortic template could lead to its improper fenestration planning and consequently compromise the surgery results. The geometry of the object being sterilized is significant to the sterilization outcome.

However, very few studies have reported on the quality of sterilization of the innovative 3D-printed objects that nowadays are often used by medical practitioners in the hospital setting. If sterilization is insufficient, microorganisms on the 3D-printed objects may easily enter into the area of human tissues, causing infection and negatively affecting the success of treatment. As sterilization is mandatory for the use of 3D-printed medical objects in the operating room, there was a need to perform a study that would evaluate the quality of sterilization of the 3D-printed aortic templates successfully used by vascular surgeons in operating rooms. Therefore, the purpose of this study was to assess the two most essential characteristics of the sterilization process of 3D medical templates, which are used in vascular surgery procedures for the treatment of aortic disorders: (i) the ability to remove microorganisms from all the surfaces of the 3D object and (ii) the ability of the 3D objects to retain their original shape, and especially, the geometry of tubular elements. In this context, the two most popular 3D printing methods, six different materials for 3D printing, and four types of sterilization used in the hospitals, were compared together in this study by performing an analysis of the deformation rate of 3D vascular model geometry in the context of the sterilization method used. The object selected for the analysis performed here was a 3D-printed tubular template of an aortic arch with its complex spatial geometry, constructed based on the CT scans of a patient suffering from thoracic aortic dissection.

## 2. Results

### 2.1. Effectiveness of Sterilization

All four sterilization methods tested in this study (autoclave 121 °C sterilization, autoclave 105 °C sterilization, plasma sterilization, and gas sterilization) were effective in destroying microorganisms. The growth of both selected indicative bacterial strains, i.e., *Geobacillus stearothermophilus* and *Bacillus atrophaeus*, was observed only in the control group.

### 2.2. Sterilization Effect on 3D Aortic Template Geometry

**Figure 1:**
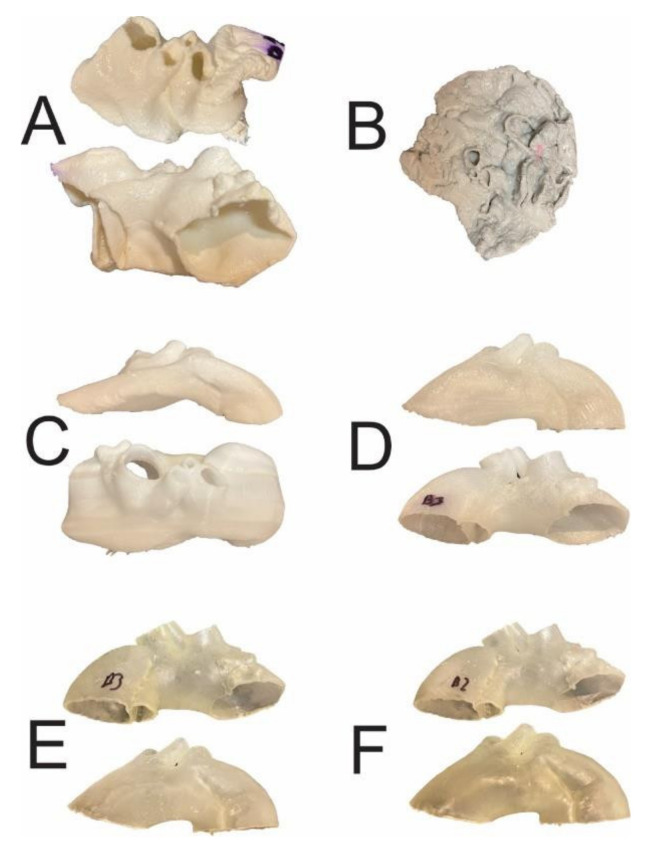
The 3D aortic templates after sterilization in 121 °C autoclave. Templates made of PLA (**A**), PETG (**B**), and PP (**C**) were affected by significant deformations, whereas those made of nylon (**D**), rigid (**E**), and flexible resins (**F**) were intact. PLA—polylactic acid; PETG—polyethylene terephthalate glycol; PP—polypropylene.

**Figure 2:**
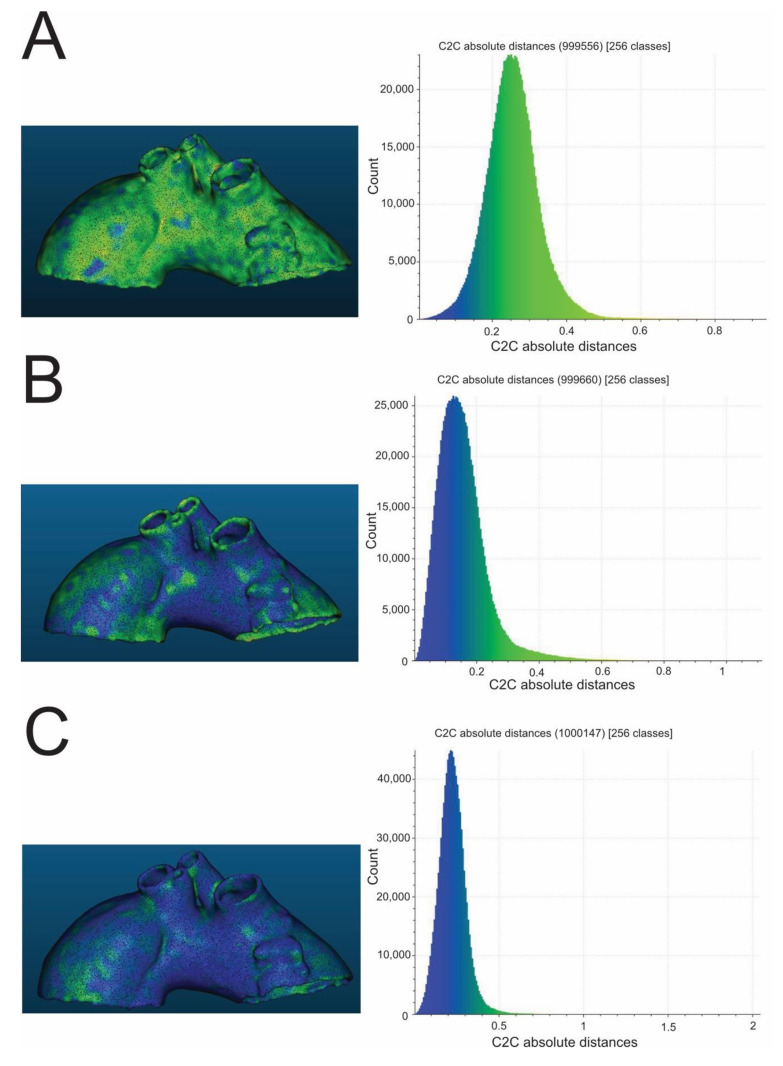
Exemplary color-coded differences (mm) between pre- and post-sterilization aortic templates and corresponding histograms. The presented aortic templates were made of PLA (**A**), PETG (**B**), and nylon (**C**). All were sterilized in a 105 °C autoclave. PLA—polylactic acid; PETG—polyethylene terephthalate glycol.

**Figure 3:**
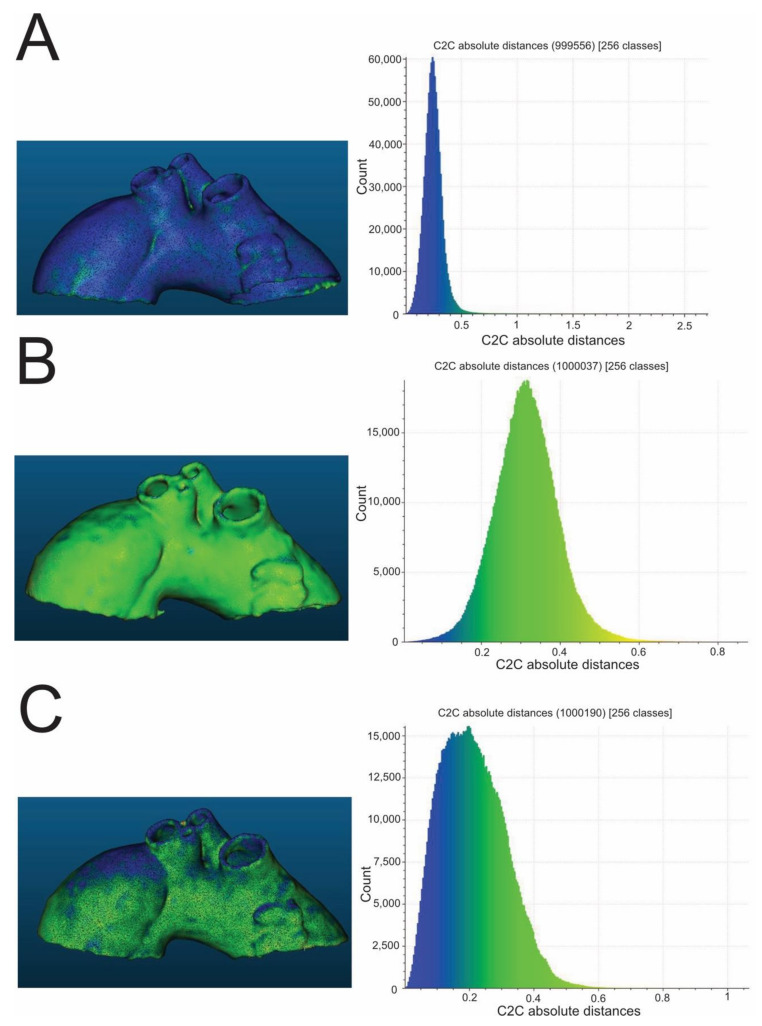
Exemplary color-coded differences (mm) between pre- and post-sterilization aortic templates and corresponding histograms. The presented aortic templates were made of PP (**A**), flexible resin (**B**), and rigid resin (**C**). All were sterilized in a 105 °C autoclave. PP—polypropylene.

High-temperature sterilization, performed with heated steam in an autoclave at 121 °C, caused macroscopically detectable, significant deformation of the aortic templates made of PLA, polyethylene terephthalate glycol (PETG), and polypropylene (PP) (Figure 1). The rest of the compounds (nylon and rigid and flexible photopolymer resin) used to produce models tested in the study were found to be regular in the visual assessment, which was subsequently confirmed through the analysis of their scanned meshes. The results of the measurement of mean geometry differences before and after the sterilization procedure are also shown in Table 1, respectively, for different compounds. All mean differences in geometry were smaller than the original CT layer thickness, i.e., <0.6 mm. In the series of all low-temperature sterilization methods tested in this study, i.e., HPP and ETO, there was no visible change in the morphology of 3D aortic templates analyzed macroscopically and subsequently using geometrical mesh analysis. All mean geometry differences were smaller than 0.6 mm for PLA, PETG, PP, nylon, rigid resin, and flexible resin. Finally, the custom-based protocol for steam sterilization in an autoclave at a lower temperature, such as 105 °C for 3 h, did not distort the processed 3D aortic templates, as determined via a visual inspection and in the next step by the dedicated CloudCompare software (Figure 2 and Figure 3), with mean differences in geometry less than 0.6 mm for all materials evaluated in this study.

## 3. Discussion

The state-of-the-art use of 3D aortic models is a safe and effective strategy to improve the ultra-modern surgical method of the treatment of the vascular system for aortic dysfunction using patient-dedicated and well-tailored stent-grafts. However, the sterilization method is still not standardized and is currently under investigation and testing. There is no consensus to date on the best testing strategies to ensure the quality of 3D-printed objects after their sterilization. In this work, an innovative analysis of macro-and micro-deformations has been developed to address the limitations of different methods of sterilization used in medical practice worldwide.

High-level microbial decontamination can be achieved in two ways: using high temperatures or using low-temperature sophisticated sterilization technology. Steam-heated sterilization is performed with high temperatures ranging from 121 to 134 degrees Celsius under pressure. When selecting sterilizing methods that involve such high temperatures, it is necessary to consider the filament type used to produce the 3D-printed template. We revealed in our study that the 3D aortic models made of PLA, PETG, and PP were macroscopically considerably distorted after steam sterilization, making the alignment of their meshes in CloudCompare software unfeasible. Likewise, Shaheen et al. reported the effect of steam sterilization on some 3D-printed objects for surgery, including a surgical cutting guide for mandible reconstruction [11]. Large deformations observed after steam-heated sterilization indicate that this method is a highly unreliable decontaminating process for 3D-printed objects. In contrast to the most common filaments used for the medical applications of 3D printing, such as PLA and PETG, which were affected by the high temperatures and pressures reached during the steam heated sterilization, the other plastic components for 3D printing tested in our study, including two different resins and nylon, they were highly resistant to high temperatures, exhibiting no differences in morphology and geometry after steam-heated sterilization. Likewise, Marei et al. also demonstrated that the morphology of dental surgical guides created from resin utilizing SLA technology remained unchanged following classical autoclave sterilization [12]. According to the findings of this investigation, steam-heated sterilization is suitable for several 3D printing compounds, such as rigid and flexible resins, as well as nylon. Considering most popular 3D printing materials’ low melting temperature and transition glass temperatures (Table 2), we then investigated the two options for sterilizing 3D printed vascular templates with low-temperature-based sterilization technology. Indeed, our results showed that the preferred sterilization method for 3D aortic templates should be low-temperature sterilization, specifically using ETO or HPP. None of the morphological assays used in this study for the detection of deformation and changes in geometry showed a notable difference between the analysis of the samples before and after low-temperature (54 °C) sterilization. Similarly to our results, another group reported the better quality of surgical guides 3D-printed with PLA and PETG when sterilized in HPP [13]. They concluded that following low-temperature sterilization, the discrepancies in noted deformations were submillimeter in size and had no clinical significance for such medical applications. Altogether, the experimental results from this study corroborated the results of other studies, indicating that the low-temperature disinfection procedures did not deform the 3D-printed models and did not significantly influence the geometry of 3D-printed tubular templates resulting from potential deterioration of the physical properties of components used for medical printing.

Even though the low-temperature sterilization methods appeared suitable for 3D printing materials in our study, there were some pitfalls related to both tested techniques, such as ETO and HPP. Although the former is commonly used for sterilization, it is highly flammable, requires special equipment, and requires a lengthy procedure lasting up to 14 h to reduce tissue toxicity, which appears after this process [6]. On the other hand, the highly expensive gas plasma sterilizers are large in size thus are difficult to maintain easily in every medical center. In contrast, autoclaves are widely distributed, their price is not so high, and they are less costly to maintain than ETO or HPP sterilizers. In this regard, we additionally tested the modified sterilization protocol with 105 degrees Celsius for three hours using a classical autoclave for steam-heated sterilization. Despite being slightly lower than the standard temperature in the autoclave and a prolonged period of disinfection, the sterilized 3D-printed specimens, as well as those composed of materials with low melting temperatures, did not show any measurable deformation or structural change in their geometry.

To study the effectiveness of the tested sterilization procedures on bacterial contamination of our 3D-printed aortic templates, we followed the recommended methods to control the sterilization process with reference bacterial strains, including *Geobacillus stearothermophilus* and *Bacillus atrophaeus*, which are dedicated to separately testing the decontamination quality of both high- and low-temperature sterilization methods, respectively [6]. All experimentally-contaminated 3D tubular models sterilized during the standard and experimental procedures were negative for bacterial growth as compared to the control 3D models, which were not sterilized at all. These results confirm the sterilization feasibility of 3D-printed aortic templates that are tubular-like structures, which naturally are challenging to sterilize if contaminated with any microorganisms, especially inside the tubular structure. Recently, the effectiveness of the sterilization of spatially closed objects was addressed by Maestro et al., who performed the sterilization of 3D-printed closed cylinders into which *S. epidermidis* was introduced [14]. They achieved complete sterility only in models subjected to ETO and hot steam, whereas single bacterial colonies were detected following gas plasma sterilization. Thus, they recommend sterilizing with ETO or hot steam but not with low-temperature plasma sterilization. In contrast to their findings, all the sterilization methods of 3D tubular models of aortic templates tested in our study appeared to be equally effective at destroying germs. Future studies should focus on wider aspects of the temperature-dependent sterilization process, particularly under different low-temperature and low-pressure conditions. Due to the preliminary nature of this study, additional research should be conducted to identify the physicochemical characteristics and pyrogenicity of the 3D aortic templates.

## 4. Materials and Methods

### 4.1. Manufacturing of Three-Dimensional Aortic Templates

**Figure 4:**
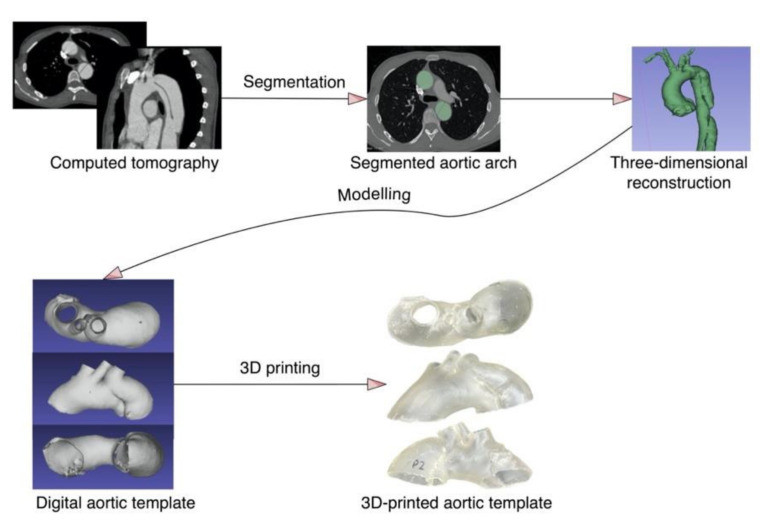
The aortic template manufacturing process.

**Figure 5:**
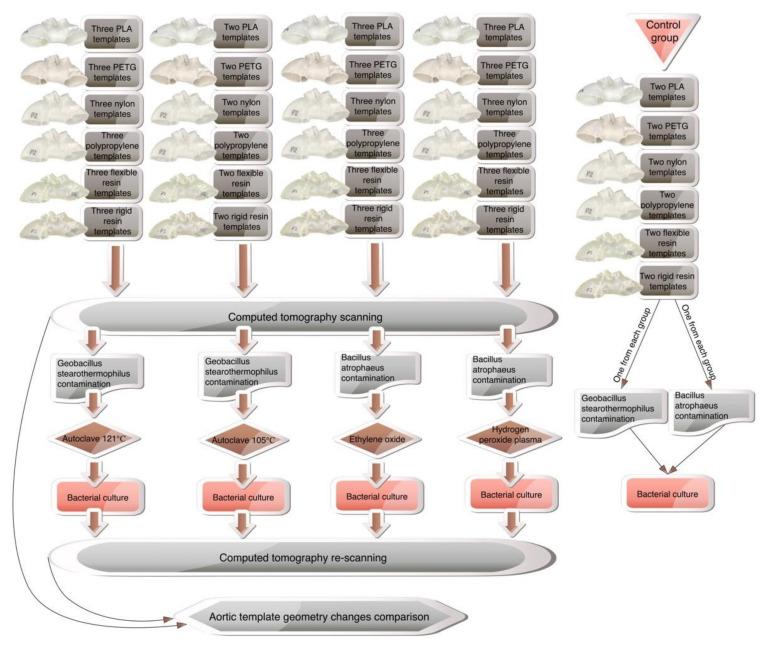
The methodology workflow.

To ensure geometric complexity, the CT of a patient with aortic arch dissection was used to create the 3D aortic template (Figure 4). A skilled vascular surgeon segmented the aorta using 3D Slicer software (version 4.11.0; https://www.slicer.org/ (accessed on 10 December 2021)) [15]. The artificial aortic wall thickness was set to 1.5 mm. A Raise3D Pro 2 printer (Raise3D, Irvine, CA, USA) was utilized for fused deposition modeling, whereas a Form 2 machine (Formlabs, Somerville, MA, USA) was used for photopolymer resins. The three-dimensional aortic arch templates were printed in polylactic acid (PLA, Raise3D, Irvine, CA, USA), nylon (Nylon PA12 filament, Fiberlogy, Brzezie, Poland), polypropylene (PP filament, Verbatim GmbH, Eschborn, Germany), polyethylene terephthalate glycol (PETG filament, Fiberlogy, Brzezie, Poland), and a rigid (standard clear resin, Formlabs, Somerville, MA, USA) and flexible photopolymer resin (UV laser Flexible resin, Photocentric, Peterborough, UK). Each material was utilized to construct 11 models, divided into four study groups as follows: autoclave 121 °C sterilization, plasma sterilization, gas sterilization, with three models in each group, and the last two models composed the group subjected to 105 °C autoclave sterilization. The two additional models were generated from each material for the establishment of the control group. The methodology’s workflow is presented in Figure 5.

### 4.2. 3D Aortic Template Scanning and Morphology Analysis

All aortic arch templates were CT-scanned before and after the sterilization process to assess changes in geometry due to sterilization. A SOMATOM Definition AS scanner (Siemens, München, Germany) with thorax settings was utilized for acquisition. All CT scans had a layer thickness of 0.6 mm. The CT-scanned models were segmented and exported as STL files using the 3D Slicer. The next stage was to compare pre-and post-sterilization meshes geometrically and dimensionally. The morphological comparison was processed in CloudCompare software.

### 4.3. Bacterial Contamination and Sterilization

*Geobacillus stearothermophilus* and *Bacillus atrophaeus* were cultured for 24 h in the liquid medium, i.e., tryptose-soy broth, at 56 °C and 37 °C, respectively. For heat sterilization, models were immersed for 15 min in *Geobacillus stearothermophilus* broth, whereas for gaseous and plasma sterilization, *Bacillus atrophaeus* broth was utilized. Additionally, a control group comprising each tested material model was contaminated (these models were not intended to be sterilized). The drying time was 24 h. The models were then sterilized using heat, HPP, and ETO.

### 4.4. Bacterial Culture

The bacterial culture was performed after the model’s sterilization and from the control group. The protocol for sampling was to wipe each 3D model in the key landmarks: all vessel ostia and from the dissected lamina, i.e., the narrowest part of both the true and false lumen. Cultures were carried out on blood agar plates and incubated in the same manner as in the previous preparation phase.

## 5. Conclusions

3D printing is gaining importance in vascular surgery, giving surgeons greater possibilities for individualized solutions in the case of patients with extraordinary aortic defects such as giant or symptomatic aneurysms or complicated aortic dissections. This work provides a smart and scientifically-proven guide to the sterilization methods of 3D-printed materials, which is suitable, for example, for medical centers involved in clinical procedures with the urgent preparation of aortic 3D templates for the treatment of patients threatened with aortic aneurysm rupture. Sterilization at high temperatures induces deformations in aortic templates composed of filaments made of PLA, PETG, and PP. However, this method is suitable for other components, including nylon or flexible and rigid resin-based models. On the other side, low-temperature sterilization can be used safely for heat-sensitive materials as we observed that HPP and ETO sterilization were appropriate for all tested components of 3D-printed vascular templates, such as PLA, PETG, PP, nylon, and both tested resins, with similar efficacy in destroying the indicative bacterial strains.

Nevertheless, if a surgical center does not have any low-temperature sterilization methods, one can consider using steam-heated sterilization at 105 degrees Celsius for three hours, which in our hands proved to be as effective as standard methods without causing deformation of the 3D aortic templates. As steam sterilization by autoclaving is an inexpensive, high-penetrating, fast-acting form of sterilization with easier accessibility in many hospitals, this readily available means of sterilization may act as a convenient and practical solution for vascular surgeons to sterilize 3D-printed aortic templates for their direct use, together with physician-modified stent-grafts, in the operating room in urgent, life-saving medical procedures.

## Figures and Tables

**Table 1:** Microbial culture results and the mean differences in geometry between pre- and post-sterilization aortic templates made of polylactic acid (PLA), polyethylene terephthalate glycol (PETG), nylon, polypropylene (PP), rigid resin, and flexible resin.

Material	Group Size	Sterilization Method	Macroscopic Damage	Mean Geometry Difference (m)	Mean SD	Microbial Culture Result
PLA	2	Autoclave 105 °C 3 h	no	0.2558 × 10^−3^	0.08 × 10^−3^	negative
PLA	3	Autoclave 121 °C 0.5 h	yes			negative
PLA	3	Ethylene oxide	no	0.3488 × 10^−3^	0.21 × 10^−3^	negative
PLA	3	Hydrogen peroxide plasma	no	0.3635 × 10^−3^	0.26 × 10^−3^	negative
PLA	2	Control group	n/a			*G. stearothermophilus*/*B. atrophaeus*
PETG	2	Autoclave 105 °C 3 h	no	0.1598 × 10^−3^	0.10 × 10^−3^	negative
PETG	3	Autoclave 121 °C 0.5 h	yes			negative
PETG	3	Ethylene oxide	no	0.2017 × 10^−3^	0.11 × 10^−3^	negative
PETG	3	Hydrogen peroxide plasma	no	0.1616 × 10^−3^	0.12 × 10^−3^	negative
PETG	2	Control group	n/a			*G. stearothermophilus*/*B. atrophaeus*
Nylon	2	Autoclave 105 °C 3 h	no	0.2256 × 10^−3^	0.08 × 10^−3^	negative
Nylon	3	Autoclave 121 °C 0.5 h	no	0.1661 × 10^−3^	0.11 × 10^−3^	negative
Nylon	3	Ethylene oxide	no	0.2798 × 10^−3^	0.10 × 10^−3^	negative
Nylon	3	Hydrogen peroxide plasma	no	0.1202 × 10^−3^	0.09 × 10^−3^	negative
Nylon	2	Control group	n/a			*G. stearothermophilus*/*B. atrophaeus*
PC	2	Autoclave 105 °C 3 h	no	0.2485 × 10^−3^	0.09 × 10^−3^	negative
PC	3	Autoclave 121 °C 0.5 h	yes			negative
PC	3	Ethylene oxide	no	0.2920 × 10^−3^	0.09 × 10^−3^	negative
PC	3	Hydrogen peroxide plasma	no	0.1055 × 10^−3^	0.06 × 10^−3^	negative
PC	2	Control group	n/a			*G. stearothermophilus*/*B. atrophaeus*
Rigid resin	2	Autoclave 105 °C 3 h	no	0.2124 × 10^−3^	0.09 × 10^−3^	negative
Rigid resin	3	Autoclave 121 °C 0.5 h	no	0.1422 × 10^−3^	0.09 × 10^−3^	negative
Rigid resin	3	Ethylene oxide	no	0.1175 × 10^−3^	0.07 × 10^−3^	negative
Rigid resin	3	Hydrogen peroxide plasma	no	0.1160 × 10^−3^	0.06 × 10^−3^	negative
Rigid resin	2	Control group	n/a			*G. stearothermophilus*/*B. atrophaeus*
Flexible resin	2	Autoclave 105 °C 3 h	no	0.3148 × 10^−3^	0.09 × 10^−3^	negative
Flexible resin	3	Autoclave 121 °C 0.5 h	no	0.1442 × 10^−3^	0.08 × 10^−3^	negative
Flexible resin	3	Ethylene oxide	no	0.3595 × 10^−3^	0.10 × 10^−3^	negative
Flexible resin	3	Hydrogen peroxide plasma	no	0.1260 × 10^−3^	0.07 × 10^−3^	negative
Flexible resin	2	Control group	n/a			*G. stearothermophilus*/*B. atrophaeus*

**Table 2:** Universal characteristics of selected 3D-printing materials used in this study.

Material	Maximum Temperature	General Features	Fabrication	Biocompatibility	Sterilization Concerns
Poly(lactic acid) (PLA)	Melting temperature: 130–180 °C; Thermal degradation: above 200 °C; Glass transition temperature: 60 °C;	Strength: High; Flexibility: Low; Water resistance: Medium; Heat resistance: Low; Chemical resistance: Low; The elastic Young’s modulus of PLA is between: 3.4–3.6 GPa; The high surface energy of PLA results in good printability, making it widely used in 3D printing	Manufactured using well-established processing technologies; PLA objects can be fabricated by 3D printing, casting, injection molding, extrusion, machining, and solvent welding; Easy for post-production	PLA belongs to the well-documented FDA-approved polymers used in the biomedical field; PLA is the most commonly used biodegradable polymer in clinical applications worldwide as it is highly biocompatible with human tissues	Not recommended for heat sterilization; Alcohol and organic solvents degrade PLA-made items; Use of beta or gamma irradiation for sterilization results in undesired reactions such as chain scissions and cyclization that lower the molecular weight of PLA and enhance its degradation rate
Polyethylene terephthalate glycol-modified (PETG)	Melting temperature: 260 °C (PETG has a higher melting point than PLA); Glass transition temperature: 85 °C;	The elastic Young’s modulus of PETG is between: 1.9–2.0 GPa; Durability: High; Strength: High; Flexibility: Low; Water resistance: High; Heat resistance: Medium; PETG is more flexible and resistant to higher temperatures than PLA; PETG has high durability, low shrinkage, and is hydrophobic	PETG is a clear amorphous thermoplastic, which is obtained from polyethylene terephthalate (PET) via copolymerization; PETG can be injection-molded, sheet-extruded, or extruded as a filament; PETG filament is designed for 3D printing in FDM technology; 3D printing with PETG is relatively easy	PETG has been reported to be a suitable polymer for tissue engineering, and it has been used in the biomedical field, for example, for prosthetic vascular grafts, due to its good mechanical properties and high biocompatibility with human tissues	PETG material is extremely resistant to chemical agents, making it perfect for use in the biomedical field; It can be sterilized easily; UV light can cause the PETG material to become weaker;
Polypropylene (PP)	Melting temperature: 160–166 °C; Glass transition temperature: 260 °C;	The elastic Young’s modulus of PP is between: 1.0–1.2 GPa; PP is liable to chain degradation from exposure to temperatures above 100 °C; PP has also been reported to biodegrade while in the human body as implantable mesh devices. PP is suitable for applications that require softness and heat resistance; PP is also highly resistant to fatigue.	Polypropylene is produced by the chain-growth polymerization of propene, and it costs less than most other synthetic fibers nowadays; PP has excellent mechanical properties, high accuracy, and repeatability; PP has a broad property profile that includes very good break-resistance, low density, and high chemical resistance, which is important in the fabrication of 3D parts	Polypropylene has been used in hernia and pelvic organ prolapse repair operations to protect the body from new hernias in the same location. A notable application was as a transvaginal mesh, used to treat vaginal prolapse and concurrent urinary incontinence; It can increase the flexibility and dimensional stability of the joint compound and reduce shrinkage and cracking when it dries.	Polypropylene at room temperature is resistant to almost all organic solvents, apart from strong oxidants; PP has good heat resistance, and it can typically withstand autoclave temperatures when correctly molded but is not recommended for repeated heat sterilization; PP is also not recommended for steam autoclaving for more than a few cycles as the material quickly loses tensile strength; PP can be damaged by long-term exposure to ultraviolet (UV) light when sterilized with UV light
Nylon	Melting temperature: 178 °C; Glass transition temperature: 70 °C;	The elastic Young’s modulus of nylon is between: 1.0–3.5 GPa; Nylon possesses excellent mechanical properties, and in particular, high impact resistance for a non-flexible filament; Nylon has good chemical resistance and filament strength	Nylon is a semi-crystalline synthetic polymer that belongs to the family of polyamides; As a thermoplastic polymer, it can be converted to fibers, films, and different shapes through melting, forming, and cooling processes	Biocompatible properties of nylon result from the presence of the amide groups in its chemical structure, which results in biomedical applications with promising potential in tissue engineering and regenerative medicine; Cells can adhere to the surface of nylon due to its hydrophilic nature and it promotes stronger mechanical adhesion between the nylon-containing medical/dental implants and human tissues	Nylon is known to be water absorbent, ultraviolet (UV) radiation-resistant, and chemical-resistant against most diluted acidic and alkaline compounds; Sterilization techniques such as ethylene oxide (ETO), gamma radiation, and steam-heated autoclaving can be applied on nylon due to its chemically inert properties

## Data Availability

The data that support the findings of this study are available from the corresponding author upon reasonable request.
